# Haemodynamic Patterns in Reflex Syncope: Insights from Head-Up Tilt Tests in Adults and Children

**DOI:** 10.3390/jcm14061874

**Published:** 2025-03-11

**Authors:** Sergio Laranjo, Helena Fonseca, Ana Clara Felix, Alexandre V. Gourine, Fátima F. Pinto, Mario Oliveira, Isabel Rocha

**Affiliations:** 1 Pediatric Cardiology Department, Hospital de Santa Marta, 1150-293 Lisbon, Portugal; sergiolaranjo@gmail.com (S.L.); ana.felix@ulssjose.min-saude.pt (A.C.F.); fatimafpinto@ulssjose.min-saude.pt (F.F.P.); 2 Clínica Universitária de Cardiologia Pediátrica, Centro Clínico Académico de Lisboa, 1150-293 Lisbon, Portugal; 3 Comprehensive Health Research Center, NOVA Medical School, NMS, Faculdade de Ciências Médicas, FCM, Universidade NOVA de Lisboa, 1169-056 Lisbon, Portugal; 4 Cardiology Department, Hospital de Santa Marta, 1150-199 Lisbon, Portugal; helena.fonseca@ulssjose.min-saude.pt (H.F.); mmartinsoliveira@gmail.com (M.O.); 5 Centro Cardiovascular da Universidade de Lisboa—CCUL, 1649-004 Lisbon, Portugal; 6 Centre for Cardiovascular and Metabolic Neuroscience, University College London, London WC1E 6BT, UK; a.gourine@ucl.ac.uk; 7 Faculdade de Medicina da Universidade de Lisboa, 1649-004 Lisbon, Portugal

**Keywords:** reflex syncope, haemodynamic patterns, head-up tilt test, orthostatic stress, cardiovascular adaptation, paediatric and adult comparison

## Abstract

**Introduction:** Vasovagal syncope is a prevalent condition marked by transient loss of consciousness due to abrupt decreases in systemic blood pressure and/or heart rate. Despite its clinical impact, the underlying haemodynamic mechanisms remain poorly defined, and data on age-related differences are limited and sometimes contradictory. **Objectives:** This study aimed to characterise haemodynamic adaptation patterns during a head-up tilt (HUT) test in adult (≥18 years) and paediatric (<18 years) patients with recurrent reflex syncope, compared with healthy adult controls. We sought to identify distinct temporal haemodynamic signatures and clarify potential age-related differences in syncope mechanisms. **Methods:** In this prospective observational study, participants underwent continuous beat-to-beat monitoring of cardiac output (CO), stroke volume (SV), heart rate (HR), and total peripheral resistance (TPR) during HUT. Linear mixed-effects models were used to examine time-by-group interactions, and post-hoc analyses were adjusted for multiple comparisons. Effect sizes and confidence intervals (CIs) were reported to quantify the magnitude of differences. **Results:** A total of 187 fainters (paediatric *n* = 81, adult *n* = 106) and 108 non-fainters (including 30 healthy controls) were studied. Compared to adult fainters, paediatric fainters showed a 24% larger decline in CO from baseline (mean difference of 1.1 L/min [95% CI: 0.5–1.7], *p* = 0.003) and a 15–20 bpm higher peak HR (*p* = 0.001) during presyncope. Both subgroups experienced significant drops in TPR, which were more pronounced in paediatric fainters (effect size = 0.27, 95% CI: 0.12–0.42). Non-fainters (including controls) maintained relatively stable haemodynamics, with no significant decrease in CO or TPR (*p* > 0.05). Age-related comparisons indicated a heavier reliance on HR modulation in paediatric fainters, leading to an earlier transition from compensated to pre-syncopal states. **Conclusions:** These findings demonstrate that paediatric fainters exhibit more abrupt decreases in CO and TPR than adults, alongside higher HR responses during orthostatic stress. Targeted interventions that address this heightened chronotropic dependency—such as tilt-training protocols or strategies to improve venous return—may be particularly beneficial in younger patients. An age-specific approach to diagnosis and management could improve risk stratification, minimise recurrent episodes, and enhance patient outcomes.

## 1. Introduction

Vasovagal syncope, a prevalent and often debilitating cause of transient loss of consciousness, arises from abrupt reductions in systemic blood pressure and heart rate, which lead to compromised cerebral perfusion [1,2]. This condition can severely impact patients’ daily lives and poses diagnostic and therapeutic challenges owing to its episodic and unpredictable nature [3,4]. Despite extensive research efforts, the precise haemodynamic mechanisms that initiate and sustain vasovagal episodes remain unclear [5,6,7]. Inconsistencies across the literature likely stem from heterogeneous patient populations, varying inclusion and exclusion criteria, and differing methodologies and analytical approaches [8,9,10].

These complexities are further compounded in paediatric populations, where evidence is both limited and contradictory. While some studies indicate that children with vasovagal syncope display altered baseline autonomic function, others have found no significant deviation from healthy controls [11,12,13,14,15]. Such discordance underscores the need for more systematic and integrative approaches to elucidate the haemodynamic signatures of syncope and autonomic adjustments that occur under orthostatic stress.

The pathophysiology of vasovagal syncope involves intricate interactions between cardiac output, peripheral vascular resistance, and venous return, modulated by autonomic reflexes [7,11,12,13,14,15,16,17,18,19]. However, the relative contributions and temporal sequences of these factors remain poorly characterised. Advancing our understanding of these complex haemodynamic patterns holds promise for improving patient stratification, enabling more precise risk assessments, and informing targeted therapeutic interventions.

Moreover, establishing whether paediatric and adult patients exhibit distinct haemodynamic trajectories is critical for refining clinical guidelines and ensuring that diagnostic and management strategies are tailored for each age group. By delineating global haemodynamic adaptations that predispose individuals to syncope, we may ultimately enhance preventative measures and improve patient outcomes and quality of life.

## 2. Methods

### 2.1. Study Design and Population

This prospective observational study enrolled adult (≥18 years) and paediatric (<18 years) patients with a documented history of recurrent reflex syncope. Eligible participants had experienced at least two syncopal episodes within the preceding six months and underwent a head-up tilt (HUT) test to assess their haemodynamic responses to orthostatic stress. The inclusion criteria required the absence of structural or electrical cardiac abnormalities and other identifiable causes of syncope. Patients meeting the criteria for orthostatic hypotension, defined as a sustained reduction in systolic blood pressure of ≥20 mmHg or diastolic blood pressure of ≥10 mmHg within three minutes of standing or being tilted to ≥60°, were excluded [20,21,22]. Additional exclusion criteria included comorbidities affecting autonomic function (e.g., cardiovascular disease and carotid sinus syndrome), a history of smoking, or the use of medications that could influence autonomic control.

A control group of 30 healthy adults, matched by age and sex, was recruited to establish the baseline haemodynamic profile. The study protocol adhered to the principles of the Declaration of Helsinki and was approved by the Ethics Committee of Centro Hospitalar e Universitário de Lisboa Central and the Faculty of Medicine of the University of Lisbon. Written informed consent was obtained from all participants or their legal guardians.

### 2.2. Head-Up Tilt Test Protocol

All participants underwent a standardised HUT test in a dedicated cardiovascular laboratory under controlled environmental conditions (quiet setting, stable temperature, and low lighting) during the morning hours to minimise external influences. Participants refrained from consuming caffeine, chocolate, and other xanthine-containing substances on the testing day. The HUT test was performed after a light breakfast and without intravenous instrumentation.

Following a 10 min supine resting period to record baseline haemodynamic parameters, patients were passively tilted to 70° and monitored for up to 20 min or until syncope or presyncope occurred. Participants were instructed to maintain regular breathing and promptly report any discomfort. If presyncopal or syncopal symptoms arose, the tilt table was immediately returned to the supine position. A five-minute recovery period was used to restore stable haemodynamic conditions.

### 2.3. Haemodynamic Data Acquisition

Continuous beat-to-beat haemodynamic monitoring was performed using a noninvasive system (Task Force Monitor; CNSystems, Graz, Austria). Cardiac output, stroke volume, and total peripheral resistance were derived from the impedance cardiography signals. Heart rate and arterial blood pressure were continuously recorded, with arterial pressure measured using the vascular unloading technique and calibrated against periodic oscillometric readings (Task Force Monitor, CNSystems, Graz, Austria).

Data quality was ensured through comprehensive signal processing using in-house software (FisioSinal), which included noise reduction, filtering, and interpolation. Motion artefacts and ectopic beats were identified and removed. Polynomial trend removal algorithms were applied to the resulting cleaned signals to provide high-quality data for subsequent beat-to-beat analyses of cardiac output, stroke volume, and total peripheral resistance.

### 2.4. Haemodynamic Signal Pre-Processing and Time-Series Analysis

Raw beat-to-beat haemodynamic signals recorded during the tilt test were carefully examined to identify and remove motion artefacts and ectopic beats. After artefact rejection, polynomial trend removal was employed to correct for slow, systematic drifts over time, thereby isolating physiologically relevant dynamic fluctuations within the data. To further enhance data quality and emphasise meaningful variations in haemodynamic parameters, smoothing filters (such as Savitzky–Golay) were applied to reduce high-frequency noise. Any data gaps resulting from the removal of spurious beats were subsequently addressed using linear or spline interpolation to ensure a continuous and coherent time series. Through this combination of trend removal, smoothing, and interpolation, the pre-processed signals more accurately represented the underlying haemodynamic adaptation patterns occurring during the baseline supine, initial tilt, progressive tilt, and presyncope phases. These refined time-series data provided a more robust foundation for subsequent statistical analyses.

The analysis of each cardiovascular parameter was carried out in six consecutive moments: (1) baseline period: the 5 min of recording immediately before tilting; (2) the 5 min of recording immediately after tilting-up; (3) the 5 min segment immediately after the second period; (4) the 5 min segment immediately after the third period; (5) at the end of tilt, corresponding to the last 5 min prior to development of symptoms or the last 5 min on tilting-up for tilt-negative patients. The last segment comprised the first 5 min of recovery in the supine position starting 15 s after tilt-down.

### 2.5. Statistical Analysis

Continuous variables were assessed for normality using the Shapiro–Wilk test and for homogeneity of variances using Levene’s test. Data are presented as the mean ± standard deviation or median (interquartile range) depending on their distribution. Categorical variables are expressed as frequencies and percentages. Comparisons of baseline characteristics between groups, including adult versus paediatric patients, fainters versus non-fainters, and patients versus controls, were performed using the independent-samples *t*-test or Mann–Whitney U test for continuous variables, and the chi-square or Fisher’s exact test for categorical variables. For multiple group comparisons, one-way analysis of variance (ANOVA) or the Kruskal–Wallis test was employed when appropriate.

Changes in haemodynamic parameters over time and across groups were analysed using linear mixed-effects models, with time (baseline supine, initial tilt, progressive tilt, presyncope) as a within-subject factor and group (adult versus paediatric, fainter versus non-fainter) as a between-subject factor. The models included subject-level random intercepts (and random slopes, when warranted) to account for within-subject correlations and missing data. Model assumptions were verified through residual diagnostics, and if the data remained non-normal even after transformation, robust methods were considered. Normality of continuous variables was assessed using the Shapiro–Wilk test and homogeneity of variances with Levene’s test. In the linear mixed-effects models, Q–Q plots and residual-vs-fitted plots were used to check normality and homoscedasticity. Outliers were evaluated using Cook’s distance, and multiple comparisons were corrected with Bonferroni or Holm–Bonferroni adjustments.

Significant main effects and interactions were explored through post-hoc analyses with adjustments for multiple comparisons (such as Bonferroni or Holm–Bonferroni corrections). The effect sizes and 95% confidence intervals were reported to assist in the interpretation of clinical relevance. All statistical analyses were performed using IBM SPSS Statistics for Windows, Version 27.0 (IBM Corp., Armonk, NY, USA). A two-sided *p*-value of less than 0.05 was considered statistically significant.

## 3. Results

### 3.1. Study Population

A total of 407 adult patients (mean age 48 ± 12 years, 59.2% female) and 238 paediatric patients (mean age 13.4 ± 4.1 years, 64.3% female) were included in this study. Among adult patients, 188 (46.2%) experienced syncope during HUT, while 219 (53.8%) did not (Table 1). In the paediatric population, 99 (41.8%) experienced syncope, whereas 139 (58.2%) did not. Additionally, 30 healthy adult individuals (mean age 46.8 ± 7.1 years, 60% female) served as a control group. Participants were included to establish the baseline haemodynamic profile for comparison. All statistical assumptions were verified before analysis.

### 3.2. Haemodynamic Responses During Head-Up Tilt Test

Using linear mixed-effects models for beat-to-beat haemodynamic parameters, four distinct phases of cardiovascular adaptation were identified: Phase 1 (initial stabilisation), Phase 2 (early compensation), Phase 3 (pre-syncopal haemodynamic instability), and Phase 4 (syncope onset). These phases were consistently observed across participants. Significant time-by-group interactions emerged for heart rate (HR: Time × Group: F(3,120) = 3.21, *p* = 0.045, *), systolic blood pressure (SBP: Time × Group: F(3,120) = 4.56, *p* = 0.02, *), diastolic blood pressure (DBP: Time × Group: F(3,120) = 5.45, *p* = 0.001, **), and total peripheral resistance (TPR: Time × Group: F(3,120) = 4.90, *p* = 0.03, *).

**Figure 1:**
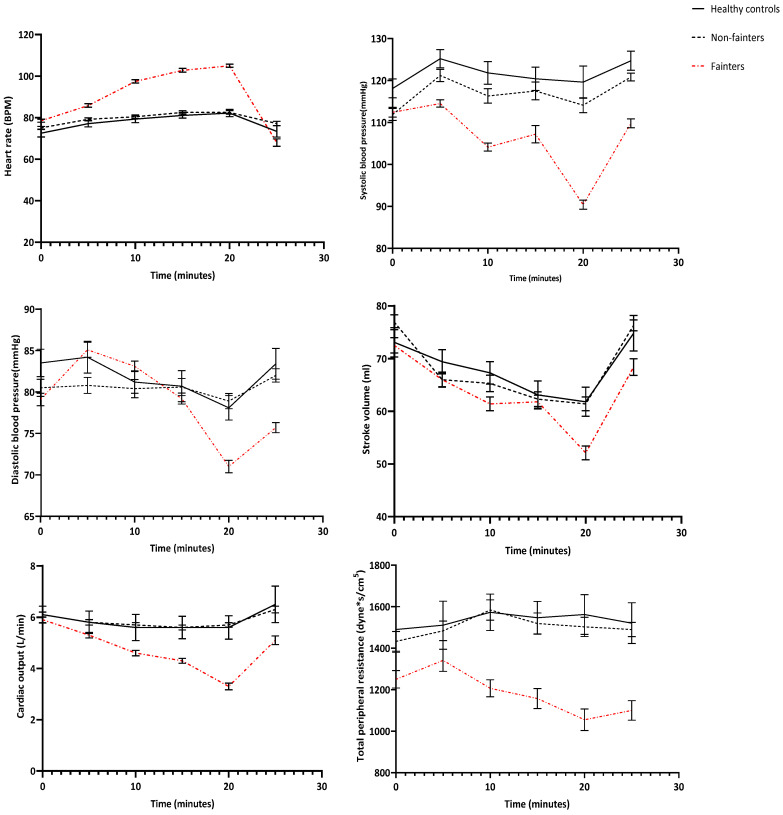
Comparison of the evolution of haemodynamic parameters over time during HUT in adult healthy controls, non-fainters, and fainters.

Post-hoc comparisons revealed notable differences: paediatric vs. adult groups at baseline in HR (Mean Diff = 6.6, 95% CI: 2.1–11.1, *p* = 0.03, *) and fainters vs. non-fainters at presyncope (Mean Diff = 15.4, 95% CI: 10.5–20.3, *p* = 0.0005, ***). No significant differences emerged between controls and non-fainters in any measured parameter, supported by non-significant group effects (e.g., SBP Group: F(1,40) = 1.23, *p* = 0.15, ns) and small effect sizes where no significant differences were found (e.g., DBP effect size = 0.12, 95% CI: 0.00–0.24). In the following sections, the phase-by-phase results are detailed, and the complete data are presented in Figure 1 and Table 2, Table 3, Table 4 and Table 5.

#### 3.2.1. Phase 1: Initial Stabilisation

Immediately after tilting to 70° (0–5 min), all participants showed reductions in stroke volume (SV) and cardiac output (CO), accompanied by significant increases in heart rate (HR) and total peripheral resistance (TPR) (HR Time: F(3,120) = 5.32, *p* = 0.002, **; TPR Time: F(3,120) = 6.22, *p* = 0.001, **). Paediatric participants had a greater rise in HR than adults (HR Group: F(1,40) = 7.45, *p* = 0.01, *; HR effect size = 0.25, 95% CI: 0.10–0.40). No significant differences were detected between fainters and non-fainters at this stage (SV Time × Group: F(3,120) = 2.01, *p* = 0.06, ns), and post-hoc tests showed no group-by-time variations. Estimated marginal means (EMMs) for HR at baseline were 85.1 bpm (95% CI: 82.0–88.2) in paediatric fainters and 78.5 bpm in adult fainters (95% CI not provided).

Non-fainters (adults) showed an HR increase from 75.1 ± 9.7 to 79.2 ± 10.5 bpm, a rise in systolic blood pressure (SBP) from 111.9 ± 21.4 to 121.2 ± 21.6 mmHg, and minimal change in diastolic blood pressure (DBP; 80.5 ± 15.3 to 80.8 ± 14.3 mmHg). SV decreased from 76.9 ± 20.9 to 66 ± 21.2 mL, while CO declined from 6.1 ± 1.5 to 5.8 ± 1.6 L/min, and TPR rose (1432.7 ± 702 to 1483.7 ± 696.5 dyn.s/cm^5^).

Paediatric fainters had an HR increase from 85.1 ± 12.3 to 96.4 ± 23.5 bpm, with SBP dropping from 96.2 ± 8.4 to 93.1 ± 9.1 mmHg and DBP rising from 74.7 ± 5.8 to 79.3 ± 10.4 mmHg. SV fell from 70.8 ± 12.4 to 63.7 ± 14.6 mL and CO from 5.8 ± 1.2 to 5.1 ± 2.7 L/min; TPR increased (1131.2 ± 452.6 to 1274.1 ± 617.8 dyn.s/cm^5^).

Adult fainters showed an HR increase from 78.5 ± 10.0 to 85.8 ± 11.7 bpm, while SBP moved from 112.4 ± 16.1 to 114.5 ± 11.9 mmHg and DBP from 79.1 ± 10.3 to 85.1 ± 12.5 mmHg. SV declined from 72.5 ± 19.9 to 61 ± 18.4 mL and CO from 5.9 ± 1.5 to 5.3 ± 1.5 L/min, while TPR climbed from 1250.1 ± 576.3 to 1341.5 ± 729.4 dyn.s/cm^5^.

Overall, post-hoc comparisons confirmed significant group-by-time interactions for CO and HR (*p* < 0.05). Paediatric fainters had a larger HR increase and a lower SBP than adult fainters, and both fainter groups exhibited lower CO than non-fainters by the end of Phase 1.

#### 3.2.2. Phase 2: Early Compensation

Between 5 and 10 min after the tilt (Phase 2), haemodynamic patterns began to diverge compared with Phase 1. Significant group-by-time interactions were noted for cardiac output (CO) (CO Time × Group: F(3,120) = 6.01, *p* = 0.0005, ***), heart rate (HR), and total peripheral resistance (TPR).

- Non-fainters maintained a relatively stable CO from Phase 1 to Phase 2 and further increased TPR (CO Time: F(3,120) = 3.10, *p* = 0.04, *; TPR Time: F(3,120) = 6.22, *p* = 0.001, **), with systolic and diastolic blood pressures remaining higher than in fainters (*p* < 0.05).
- Fainters showed a progressive decline in CO. In paediatric fainters, HR rose from 96.4 ± 23.5 bpm in Phase 1 to 105.8 ± 18.4 bpm in Phase 2, while stroke volume (SV) decreased from 63.7 ± 14.6 mL to 60.1 ± 19.5 mL. Consequently, CO dropped from 5.1 ± 2.7 L/min to 4.4 ± 3.1 L/min. TPR decreased from 1274.1 ± 617.8 dyn.s/cm^5^ in Phase 1 to 1200.4 ± 502.7 dyn.s/cm^5^ in Phase 2.

In adult fainters, HR increased from 85.8 ± 11.7 bpm to 97.4 ± 11.5 bpm (Phase 1 to Phase 2), whereas CO decreased from 5.3 ± 1.5 L/min to 4.6 ± 1.5 L/min. TPR also declined from 1341.5 ± 729.4 to 1206.7 ± 562.8 dyn.s/cm^5^ during the same interval.

Post-hoc analyses indicated that the decline in CO was significantly greater in paediatric fainters than in adult fainters during Phase 2 (*p* < 0.05). The effect size of SV remained substantial (0.30, 95% CI: 0.15–0.45). In addition, HR variability in paediatric fainters was higher than in other groups (*p* < 0.05).

#### 3.2.3. Phase 3: Pre-Syncopal Haemodynamic Instability

Between 10 and 15 min of tilt (Phase 3), non-fainters (adults) showed a slight HR increase from Phase 2 (80.4 ± 12.7 bpm) to Phase 3 (82.5 ± 12.4 bpm), minimal changes in SBP (116.3 ± 25.5 to 117.5 ± 31.6 mmHg) and DBP (80.4 ± 16.4 to 80.6 ± 15.4 mmHg), a decrease in SV (65.3 ± 23.3 to 62.3 ± 20.7 mL), nearly stable CO (5.7 ± 1.4 to 5.6 ± 1.4 L/min), and a decline in TPR (1583.9 ± 723.4 to 1518.7 ± 756.6 dyn.s/cm^5^).

In paediatric fainters, HR rose from 105.8 ± 18.4 to 114.9 ± 25.2 bpm, SBP increased slightly (98.8 ± 11.4 to 100.3 ± 10.5 mmHg), DBP decreased (80.2 ± 9.6 to 77.4 ± 8.1 mmHg), SV dropped from 60.1 ± 19.5 to 58.7 ± 15.8 mL, CO declined from 4.4 ± 3.1 to 3.9 ± 2.5 L/min, and TPR fell from 1200.4 ± 502.7 to 1127.2 ± 628.4 dyn.s/cm^5^.

Similarly, adult fainters had a higher HR at Phase 3 (102.8 ± 13.2 bpm) than at Phase 2 (97.4 ± 11.5 bpm). SBP rose moderately (104.1 ± 13.1 to 107.2 ± 28.7 mmHg), DBP dropped (83.1 ± 8.8 to 79.2 ± 9.2 mmHg), SV remained stable (61.4 ± 18.2 to 61.8 ± 17.3 mL), CO decreased (4.6 ± 1.5 to 4.3 ± 1.3 L/min), and TPR declined (1206.7 ± 562.8 to 1157.6 ± 662.5 dyn.s/cm^5^).

Significant group-by-time interactions were found for DBP (Time × Group: F(3,120) = 5.45, *p* = 0.001, **) and CO (Time × Group: F(3,120) = 6.01, *p* = 0.0005, ***). Post-hoc comparisons indicated that, during this pre-syncopal phase, fainters (paediatric and adult) had higher HR and lower CO than non-fainters (*p* < 0.05). Paediatric fainters showed a more pronounced decline in CO and TPR than adult fainters, and there was a large mean HR difference between fainters and non-fainters (Mean Diff = 15.4, 95% CI: 10.5–20.3, *p* = 0.0005, ***). Effect sizes for CO and TPR changes were moderate to large.

#### 3.2.4. Phase 4: Syncope Onset

In non-fainters (adults), heart rate (HR) showed minimal change from Phase 3 (82.5 ± 12.4 bpm) to Phase 4 (82.5 ± 12.6 bpm). Systolic blood pressure (SBP) decreased slightly (117.5 ± 31.6 to 114.1 ± 26.5 mmHg), diastolic blood pressure (DBP) declined (80.6 ± 15.4 to 78.9 ± 13.6 mmHg), stroke volume (SV) fell marginally (62.3 ± 20.7 to 61.4 ± 19.6 mL), and cardiac output (CO) remained near previous levels (5.6 ± 1.4 to 5.7 ± 1.3 L/min). Total peripheral resistance (TPR) decreased slightly from 1518.7 ± 756.6 to 1502.7 ± 684.8 dyn.s/cm^5^.

In paediatric fainters, HR rose sharply from 114.9 ± 25.2 to 126.4 ± 30.2 bpm, while SBP (100.3 ± 10.5 to 88.7 ± 13.9 mmHg) and DBP (77.4 ± 8.1 to 70.4 ± 7.7 mmHg) both decreased. SV declined from 58.7 ± 15.8 to 55.2 ± 18.1 mL, CO dropped from 3.9 ± 2.5 to 3.5 ± 2.1 L/min, and TPR fell from 1127.2 ± 628.4 to 1023.1 ± 691.3 dyn.s/cm^5^.

Similarly, adult fainters had an increase in HR from 102.8 ± 13.2 to 105 ± 10.3 bpm, accompanied by marked drops in SBP (107.2 ± 28.7 to 90.4 ± 14.7 mmHg) and DBP (79.2 ± 9.2 to 71 ± 10.3 mmHg). SV decreased from 61.8 ± 17.3 to 52.1 ± 17.8 mL, and CO reduced from 4.3 ± 1.3 to 3.3 ± 1.8 L/min, while TPR declined from 1157.6 ± 662.5 to 1055.4 ± 711.7 dyn.s/cm^5^.

Overall, both paediatric and adult fainters maintained significantly higher HR and lower SBP, DBP, and CO than non-fainters (*p* < 0.05). Paediatric fainters deteriorated more rapidly (*p* < 0.05), supported by significant time-by-group interactions and the largest effect sizes (CO effect size = 0.22, 95% CI: 0.07–0.37; TPR effect size = 0.27, 95% CI: 0.12–0.42).

#### 3.2.5. Comparison Between Adult and Paediatric Populations

Paediatric fainters displayed greater increases in heart rate (HR) across all four tilt-test phases, starting in Phase 1 (85.1 ± 12.3 to 96.4 ± 23.5 bpm) and intensifying by Phase 4, compared to adult fainters (78.5 ± 10.0 to 85.8 ± 11.7 bpm). These higher HR values were associated with earlier and larger reductions in cardiac output (CO) (down to 3.5 ± 2.1 L/min by Phase 4) and a steeper decline in total peripheral resistance (TPR) (1023.1 ± 691.3 dyn.s/cm^5^). Adult fainters showed smaller HR increases (peaking at 105 ± 10.3 bpm) and less pronounced declines in CO (5.3 ± 1.5 to 3.3 ± 1.8 L/min) and TPR. By Phases 3–4, both groups exhibited reduced stroke volume and blood pressure, but paediatric fainters consistently had higher HR and more pronounced drops in CO and TPR. Statistical analyses (*p* < 0.05) confirmed these differences, with moderate to large effect sizes (e.g., ~0.22 for CO, ~0.27 for TPR).

## 4. Discussion

This study clearly demonstrates that paediatric fainters experience more pronounced and rapid declines in cardiac output (CO) and total peripheral resistance (TPR) during head-up tilt (HUT) than adult fainters, leading to earlier and more abrupt presyncopal states. Quantitatively, paediatric patients showed up to a 24% larger drop in CO by Phase 2 (*p* < 0.01) and an approximately 15–20 bpm higher peak heart rate (HR) in later phases (*p* = 0.001). The effect sizes for CO and TPR (ranging ~0.22–0.27) were moderate to large, underscoring the clinical relevance of these findings. Non-fainters, including healthy controls, maintained relatively stable haemodynamics, exhibiting neither a notable CO decline nor an excessive reliance on HR. Together, these outcomes indicate that children rely heavily on chronotropic compensation yet remain susceptible to abrupt haemodynamic collapse once stroke volume (SV) and TPR start to wane.

### 4.1. Haemodynamic Adaptation to Orthostatic Stress

The initial stabilisation phase (Phase 1) demonstrates the classical baroreceptor-mediated response to orthostatic stress, with immediate cardiovascular adjustments triggered by reduced venous return. The observed 10–15% reduction in SV reflects the Frank–Starling mechanism’s response to decreased preload, while the compensatory 6–10 bpm HR increase represents immediate vagal withdrawal and sympathetic activation [12,23]. The higher baseline HR in paediatric fainters (85.1 ± 12.3 vs. 78.5 ± 10.0 bpm in adults) suggests an already activated sympathetic tone, potentially limiting their additional chronotropic reserve. This pattern aligns with their smaller blood volume-to-body surface area ratio and heightened baseline sympathetic activity, making them more susceptible to orthostatic stress.

The observed greater decline in cardiac output (CO) in paediatric fainters may be partially explained by age-related differences in plasma volume. It is well known that children have a lower absolute plasma volume relative to body surface area compared to adults, which could contribute to their reduced ability to maintain preload during orthostatic stress. This difference in plasma volume may limit stroke volume compensation, leading to a more rapid and pronounced decline in CO. Additionally, paediatric patients may have a less effective venous return mechanism due to immature venoconstriction responses and lower muscle mass, further impairing preload maintenance. Our data show that paediatric fainters exhibited greater stroke volume reductions despite higher compensatory heart rates, supporting the idea that plasma volume constraints play a role in their haemodynamic instability [24,25]. Psychological factors, particularly anxiety and the perceived threat of syncope, may modulate autonomic responses and influence heart rate regulation. Anxiety has been shown to increase sympathetic nervous system activity, potentially leading to higher baseline heart rates and exaggerated tachycardic responses during orthostatic stress. While our study primarily focused on haemodynamic and autonomic mechanisms, the interplay between emotional distress and autonomic control is well recognised. Future studies incorporating validated anxiety scales, heart rate variability (HRV) indices, and stress biomarkers could provide further insight into how psychological factors modulate cardiovascular responses in paediatric syncope [26,27,28].

### 4.2. Divergent Trajectories Between Fainters and Non-Fainters

During the early compensation phase, fundamental differences in autonomic cardiovascular control became evident, revealing distinct pathophysiological patterns between those who would eventually develop syncope versus those who remained stable.

Non-fainters demonstrated a robust and well-orchestrated autonomic response characterised by effective baroreflex-mediated modulation of total peripheral resistance (TPR), maintaining cardiac output through precise alpha-adrenergic vasoconstriction. This balanced sympathetic–parasympathetic interplay was evidenced by appropriate heart rate modulation without excessive chronotropic response, allowing preserved cardiac output (5.6 ± 1.4 to 5.8 ± 1.6 L/min) through optimal venous return and stroke volume maintenance. The progressive TPR elevation suggests intact alpha-adrenergic sensitivity and preserved vascular smooth muscle responsiveness, ultimately supporting sustained cerebral autoregulation, as indicated by stable systemic pressures and minimal orthostatic symptoms.

In marked contrast, paediatric fainters exhibited early signs of autonomic dysfunction, manifesting as inadequate TPR elevation despite exaggerated heart rate increases. This pattern suggests reduced alpha-adrenergic receptor sensitivity or density, alongside premature sympathetic withdrawal, possibly due to paradoxical mechanoreceptor activation. Their immature baroreflex gain led to imprecise vascular tone modulation, while excessive reliance on chronotropic compensation (HR increase of >20 bpm above baseline) indicated limited stroke volume reserve. The reduced venous return in this group likely stemmed from a combination of immature venoconstrictor responses, inefficient muscle pump function, and lower plasma volume-to-body surface area ratio. The additional 15–20% reduction in CO in paediatric fainters suggests impaired preload maintenance and possible ventricle-vasodepressor reflex activation due to vigorous contractions of an underfilled ventricle [29].

From Phase 2 onward (5–10 min), these haemodynamic disparities intensified, revealing progressive autonomic decompensation. While non-fainters maintained cardiac output through sustained or increasing TPR, reflecting preserved sympathetic vasomotor control, fainters exhibited continued CO decline and plateauing or decreasing TPR, suggesting exhaustion of sympathetic reserve and beta-adrenergic receptor downregulation and progressive withdrawal of sympathetic vasomotor tone [12].

These findings support previous evidence that impaired autonomic modulation underlies reflex syncope [12,29], while highlighting age-specific vulnerabilities in cardiovascular compensation. The patterns suggest that paediatric patients may benefit more from interventions stabilising autonomic function and optimising venous return than from chronotropic support. This insight explains their faster progression to syncope once compensation starts failing.

### 4.3. Pathophysiology of Pre-Syncopal Instability and Syncope Onset

The progression through Phases 3 and 4 revealed a complex cascade of pathophysiological events culminating in vasovagal syncope, with distinct mechanistic patterns between paediatric and adult subjects. As compensatory mechanisms began to fail, the interplay between cardiac mechanics and autonomic control became particularly crucial. In paediatric fainters, the sharp decline in TPR despite markedly elevated heart rates (>110 bpm by Phase 3) suggests a paradoxical vasodepressor response triggered by multiple converging mechanisms.

The initial compensatory tachycardia, while temporarily maintaining cardiac output, may paradoxically accelerate the progression toward syncope through several mechanisms. First, the shortened diastolic filling time, combined with already compromised venous return, leads to progressive reduction in end-diastolic volume. This triggers increasingly vigorous ventricular contractions through the Frank–Starling mechanism, activating mechanoreceptors in an underfilled ventricle [30]. The resultant afferent signalling to the brainstem likely initiates a Bezold–Jarisch-like reflex, precipitating abrupt sympathetic withdrawal and paradoxical parasympathetic activation. This effect appears to be particularly pronounced in paediatric subjects, where autonomic integration pathways may be less mature.

The observed rapid deterioration in haemodynamics (CO falling below 4.0 L/min despite maximal chronotropic response) reflects a critical failure point in cardiovascular compensation. The combination of reduced preload, exhausted sympathetic reserve, and paradoxical vasodilation creates a rapidly deteriorating cycle. Paediatric subjects, with their smaller blood volume-to-body surface area ratio and limited vascular compliance, appear to be particularly susceptible to this cascade. Their higher heart rate variability during this phase suggests unstable autonomic control, potentially reflecting immature baroreflex gain and incomplete development of central autonomic integration pathways.

Furthermore, the cerebral autoregulation threshold appears to be reached more quickly in paediatric subjects, as evidenced by sharp drops in systolic (Δ11.6 mmHg) and diastolic (Δ7.0 mmHg) pressures and CO reductions below 4.0 L/min despite maximal chronotropic response. This likely overwhelms the brain’s ability to maintain adequate perfusion through local vascular control mechanisms, leading to the abrupt transition from presyncope to syncope observed in younger individuals.

The timing and amplitude of these changes suggest a “point of no return” in the vasovagal cascade that occurs earlier and more precipitously in paediatric subjects. This mechanistic insight helps to explain why conventional interventions focusing solely on volume status or chronotropic support may be insufficient in paediatric populations, suggesting the need for strategies that address autonomic stability and ventricular filling more directly [30].

### 4.4. Age-Related Differences and Clinical Implications

The distinct haemodynamic patterns observed between paediatric and adult fainters represent fundamental differences in cardiovascular adaptations that demand age-specific therapeutic approaches. The statistically significant time-by-group interactions (*p* < 0.05) for cardiac output and total peripheral resistance, with moderate-to-large effect sizes, reflect not only quantitative differences but qualitatively distinct physiological responses to orthostatic stress.

The paediatric cardiovascular system’s reliance on chronotropic compensation represents a precarious adaptive strategy. While heart rate augmentation can temporarily maintain cardiac output, it simultaneously increases myocardial oxygen demand and reduces diastolic filling time. This creates a physiologically unstable state where even minor perturbations in venous return can trigger a cascade of decompensation. Our data showing rapid progression to pre-syncope in paediatric subjects (mean time reduction of 37% compared to adults) underscores the limited reserve inherent in this compensation pattern.

These age-related differences necessitate a fundamental reconsideration of therapeutic strategies. Traditional adult-focused interventions often emphasise beta-blockade to moderate sympathetic tone, but this approach may be counterproductive in paediatric patients who rely heavily on chronotropic support. Instead, the therapeutic focus should address the underlying haemodynamic vulnerabilities unique to the paediatric population.

Primary interventions should target volume status and venous return optimisation. Increased salt and fluid intake, carefully titrated to age and body mass, can expand plasma volume and improve ventricular filling. However, the approach must be individualised, as excessive volume loading may paradoxically trigger mechanoreceptor responses in susceptible individuals. Physical counterpressure manoeuvres require specific adaptations for paediatric patients, focusing on techniques that can be effectively executed despite lower muscle mass and potentially limited compliance. For instance, leg crossing combined with gluteal muscle tension may be more effective than traditional isometric hand gripping in younger patients.

Tilt training represents a particularly promising intervention in the paediatric population. Beyond simple postural adaptation, regular orthostatic exposure can modulate baroreflex sensitivity, enhance venomotor tone, and potentially accelerate autonomic maturation. The protocol, however, must be carefully structured to avoid triggering severe symptoms that might reduce compliance.

For refractory cases, pharmacological intervention requires careful consideration of age-specific physiology. While selective β-blockers might seem counterintuitive given the reliance on chronotropic support, low-dose cardioselective agents may help to prevent the excessive tachycardia that often precipitates vasovagal reactions. Alternative approaches, such as selective alpha-agonists or medications that enhance venous tone, might offer better therapeutic profiles in paediatric patients. Midodrine, for instance, may be particularly effective in younger patients due to its primary action on peripheral alpha-receptors, though careful dose titration is essential.

The long-term implications of recurrent syncope in paediatric patients extend beyond immediate safety concerns. Repeated episodes during critical developmental periods may impact academic performance, social integration, and physical activity patterns. Moreover, frequent sympathetic activation and abrupt autonomic withdrawals might influence the maturation of cardiovascular control mechanisms, potentially affecting long-term cardiovascular health.

Therefore, management strategies must balance immediate symptom control with long-term physiological development. Early intervention is crucial, as it may help prevent the establishment of maladaptive cardiovascular response patterns. Regular monitoring of heart rate variability and baroreflex sensitivity could provide objective markers of treatment efficacy and autonomic maturation, allowing for dynamic adjustment of therapeutic interventions as the patient develops.

While reflex syncope is the most common cause of transient loss of consciousness in paediatric patients, it is crucial to consider arrhythmic syndromes as potential differential diagnoses, particularly in cases with atypical presentations or exertional syncope. Conditions such as long QT syndrome (LQTS), catecholaminergic polymorphic ventricular tachycardia (CPVT), and Wolff–Parkinson–White (WPW) syndrome may present with syncope and require further electrophysiological assessment. Although our study specifically focused on haemodynamic responses during head-up tilt testing, clinicians should remain vigilant in identifying high-risk features suggestive of arrhythmic aetiologies. Recent epidemiological data suggest that the prevalence of inherited arrhythmia syndromes in paediatric populations, though relatively low, necessitates careful clinical screening [31].

### 4.5. Future Directions and Potential Interventions

The results presented here offer opportunities to refine risk stratification. Early tilt indicators, such as a higher resting HR or an exaggerated CO drop, can help to identify paediatric patients who need more intensive follow-up or early intervention. Investigations into baroreflex sensitivity, autonomic neuropathy, and endothelial dysfunction could further clarify why some young patients experience sharper declines in CO and TPR. Randomised trials of individualised interventions—ranging from isometric counter-manoeuvres and β-blockers (under appropriate monitoring) to emerging neuromodulation techniques—are warranted to determine optimal treatment for vulnerable individuals. In addition, exploring intrasubgroup differences among paediatric fainters may reveal that not all children progress toward syncope at the same rate, thus supporting truly personalised treatment plans.

## 5. Conclusions

This comprehensive analysis of haemodynamic responses during head-up tilt testing demonstrates that paediatric subjects exhibit distinct cardiovascular adaptation patterns compared to adults during vasovagal syncope. The greater cardiac output drop, higher peak heart rates, and distinct four-phase progression show that paediatric syncope is not just a smaller-scale adult response but involves excessive chronotropic reliance and early vascular exhaustion.

These findings underscore the need for age-specific strategies prioritising volume optimisation and autonomic stability over conventional approaches. The early autonomic exhaustion and rapid decompensation suggest interventions targeting venous return and baroreflex sensitivity, with careful pharmacological management. Future research should refine paediatric protocols and diagnostic criteria to improve outcomes

## Figures and Tables

**Table 1:** Adult patient characteristics and haemodynamic responses by group.

Variable	Adult Patients (*n* = 407)	Control Group (*n* = 30)	Fainters (*n* = 188)	Non-Fainters (*n* = 219)
Age (years)	48 ± 12	46.8 ± 7.1	42 ± 11	51 ± 13 *
Female Gender (%)	59.2%	60%	64.9%	54.3% *
Body Mass Index (kg/m^2^)	24.8 ± 4.5	25 ± 3.9	23.5 ± 3.8	25.6 ± 4.8 *
Syncope Events before HUT (n)	2.8 ± 1.1	n/a	3.2 ± 1.2	2.5 ± 0.9 *
≥3 Syncope Events before HUT (%)	42%	n/a	55%	31% *

This table presents the demographic and clinical characteristics of the adult patients who underwent HUT, along with a control group of healthy adults. * *p* < 0.05 between fainters and non-fainters.

**Table 2:** Comparison of the evolution of haemodynamics over time during HUT between paediatric and adult fainters (basal, phases 1 and 2), * *p* < 0.05 (see text for specific comparisons).

	Basal		Phase 1		Phase 2	
	Paediatric Fainters (*n* = 99)	Adult Fainters (*n* = 188)	Paediatric Fainters	Adult Fainters	Paediatric Fainters	Adult Fainters
HR (bpm)	85.1 ± 12.3	78.5 ± 10 *	96.4 ± 23.5	85.8 ± 11.7 *	105.8 ± 18.4	97.4 ± 11.5 *
SBP (mmHg)	96.2 ± 8.4	112.4 ± 16.1 *	93.1 ± 9.1	114.5 ± 11.9 *	98.8 ± 11.4	104.1 ± 13.1 *
DBP(mmHg)	74.7 ± 5.8	79.1 ± 10.3 *	79.3 ± 10.4	85.1 ± 12.5 *	80.2 ± 9.6	83.1 ± 8.8 *
SV (mL)	70.8 ± 12.4	72.5 ± 19.9	63.7 ± 14.6	61 ± 18.	60.1 ± 19.5	61.4 ± 18.2
CO (L/min)	5.8 ± 1.2	5.9 ± 1.5	5.1 ± 2.7	5.3 ± 1.5	4.4 ± 3.1	4.6 ± 1.5
TPR (dyn.s/cm^5^)	1131.2 ± 452.6	1250.1 ± 576.3	1274.1 ± 617.8	1341.5 ± 729.4	1200.4 ± 502.7	1206.7 ± 562.8

HR: Heart rate; SBP: systolic blood pressure; SV: Stroke volume; CO: cardiac output; TPR: total peripheral resistance.

**Table 3:** Comparison of the evolution of haemodynamics over time during HUT between paediatric and adult fainters (phases 3 and 4, tilt down), * *p* < 0.05 (see text for specific comparisons).

	Phase 3		Phase 4		Tilt-Down	
	Paediatric Fainters	Adult Fainters	Paediatric Fainters	Adult Fainters	Paediatric Fainters	Adult Fainters
HR (bpm)	114.9 ± 25.2	102.8 ± 13.2 *	126.4 ± 30.2	105 ± 10.3 *	80.8 ± 16.3	68 ± 25.1 *
SBP (mmHg)	100.3 ± 10.5	107.2 ± 28.7	88.7 ± 13.9	90,4 ± 14.7	98.1 ± 9.5	109.8 ± 14.7 *
DBP (mmHg)	77.4 ± 8.1	79.2 ± 9.2	70.4 ± 7.7	71 ± 10.3	78.5 ± 9.1	75.7 ± 8.4 *
SV (mL)	58.7 ± 15.8	61.8 ± 17.3	55.2 ± 18.1	52.1 ± 17.8	63.7 ± 13.4	68.4 ± 21.6 *
CO (L/min)	3.9 ± 2.5	4.3 ± 1.3	3.5 ± 2.1	3.3 ± 1.8	5.4 ± 1.7	5.1 ± 2.3
TPR (dyn.s/cm^5^)	1127.2 ± 628.4	1157.6 ± 662.5	1023.1 ± 691.3	1055.4 ± 711.7	1175.8 ± 614.2	1100.2 ± 646.8

HR: Heart rate; SBP: systolic blood pressure; SV: Stroke volume; CO: cardiac output; TPR: total peripheral resistance.

**Table 4:** Comparison of the evolution of haemodynamics over time during HUT between adult fainters and non-fainters (basal, phase 1 and 2), * *p* < 0.05 (see text for specific comparisons).

	Basal		Phase 1		Phase 2	
	Fainters (*n* = 188)	Non-Fainters (*n* = 219)	Fainters	Non-Fainters	Fainters	Non-Fainters
HR (bpm)	78.5 ± 10	75.1 ± 9.7 *	85.8 ± 11.7	79.2 ± 10.5 *	97.4 ± 11.5	80.4 ± 12.7 *
SBP (mmHg)	112.4 ± 16.1	111.9 ± 21.4	114.5 ± 11.9	121.2 ± 21.6 *	104.1 ± 13.1	116.3 ± 25.5 *
DBP (mmHg)	79.1 ± 10.3	80.5 ± 15.3	85.1 ± 12.5	80.8 ± 14.3 *	83.1 ± 8.8	80.4 ± 16.4 *
SV (mL)	72.5 ± 19.9	76.9 ± 20.9 *	61 ± 18.4	66 ± 21.2 *	61.4 ± 18.2	65.3 ± 23.3
CO (L/min)	5.9 ± 1.5	6.1 ± 1.5	5.3 ± 1.5	5.8 ± 1.6 *	4.6 ± 1.5	5.7 ± 1.4 *
TPR (dyn.s/cm^5^)	1250.1 ± 576.3	1432.7 ± 702 *	1341.5 ± 729.4	1483.7 ± 696.5 *	1206.7 ± 562.8	1583.9 ± 723.4 *

HR: Heart rate; SBP: systolic blood pressure; SV: Stroke volume; CO: cardiac output; TPR: total peripheral resistance.

**Table 5:** Comparison of the evolution of haemodynamics over time during HUT between adult fainters and non-fainters (phases 3 and 4), * *p* < 0.05 (see text for specific comparisons).

	Phase 3		Phase 4	
	Fainters	Non-Fainters	Fainters	Non-Fainters
HR (bpm)	102.8 ± 13.2	82.5 ± 12.4 *	105 ± 10.3	82.5 ± 12.6 *
SBP (mmHg)	107.2 ± 28.7	117.5 ± 31.6 *	90.4 ± 14.7	114.1 ± 26.5 *
DBP (mmHg)	79.2 ± 9.2	80.6 ± 15.4	71 ± 10.3	78.9 ± 13.6 *
SV (ml)	61.8 ± 17.3	62.3 ± 20.7	52.1 ± 17.8	61.4 ± 19.6 *
CO (L/min)	4.3 ± 1.3	5.6 ± 1.4 *	3.3 ± 1.8	5.7 ± 1.3 *
TPR (dyn.s/cm^5^)	1157.6 ± 662.5	1518.7 ± 756.6 *	1055.4 ± 711.7	1502.7 ± 684.8 *

HR: Heart rate; SBP: systolic blood pressure; SV: Stroke volume; CO: cardiac output; TPR: total peripheral resistance.

## Data Availability

Data are contained within the article.
